# Assessing potential countermeasures against the dengue epidemic in non-tropical urban cities

**DOI:** 10.1186/s12976-016-0039-0

**Published:** 2016-04-12

**Authors:** Hiroki Masui, Itsuki Kakitani, Shumpei Ujiyama, Kazuyoshi Hashidate, Masataka Shiono, Kazue Kudo

**Affiliations:** Undergraduate School of Informatics and Mathematical Science, Faculty of Engineering, Kyoto University, Yoshida-Honmachi, Sakyo-ku, Kyoto, 606-8501 Japan; Department of Global Agricultural Sciences, Graduate School of Agricultural and Life Sciences, The University of Tokyo, 1-1-1, Yayoi, Bunkyo-ku, Tokyo, 113-8657 Japan; Department of Value and Decision Science, Tokyo Institute of Technology, 2-12-1 Ookayama, Meguro-ku, Tokyo, 152-8550 Japan; Fukoku Mutual Life Insurance Company, 2-2, Uchisaiwaicho 2-chome, Chiyoda-ku, Tokyo, 100-0011 Japan; Gunma Prefectural Institute of Public Health and Environmental Sciences, 378 Kamioki-Machi, Maebashi city, Gunma, 371-0052 Japan; Department of Computer Science, Ochanomizu University, 2-1-1 Ohtsuka, Bunkyo-ku, Tokyo, 112-8610 Japan

**Keywords:** Dengue mathematical model, Sensitivity analysis, Core population

## Abstract

**Background:**

Dengue is a common mosquito-borne viral disease epidemic especially in tropical and sub-tropical regions where water sanitation is not substantially controlled. However, dengue epidemics sometimes occur in non-tropical urban cities with substantial water sanitary control. Using a mathematical model, we investigate what conditions can be important for a dengue epidemic to occur in an urban city such as Tokyo, where vectors are active only in summer and there are little number of vectors around hosts.

**Methods:**

The model, which is a modified Ross-Macdonald model, consists of two sets of host-vector compartments. The two sets correspond to high-risk and low-risk areas, and only hosts can move between them. Assuming that mosquitoes have constant activity for only 90 days, we assess five potential countermeasures: (1) restricted movement between the two areas, (2) insecticide application, (3) use of repellents, (4) vector control, and (5) isolation of the infected.

**Results:**

The basic reproduction number *R*_0_ and the cumulative number of infected hosts for 90 days are evaluated for each of the five countermeasures. In the cases of Measures 2–5, the cumulative number of the infected for 90 days can be reduced substantially for small *R*_0_ even if *R*_0_>1. Although *R*_0_ for Measure 1 monotonically decreases with the mobility rates, the cumulative number of the infected for 90 days has a maximum at a moderate mobility rate. If the mobility rate is sufficiently small, the restricted movement effectively increases the number density of vectors in the high-risk area, and the epidemic starts earlier in the high-risk area than in the low-risk one, while the growth of infections is slow.

**Conclusions:**

Measures 2–5 are more or less effective. However, Measure 1 can have the opposite effect, depending on the mobility rates. The restricted movement results in the formation of a kind of core population, which can promote the epidemic in the entire population.

## Background

Dengue is a mosquito-borne viral infection. The number of the global incidence of dengue has grown dramatically in recent years, and 3900 million people, in 128 countries are under the risk of infection [1, 2]. The main vector for transmission of the dengue virus is *Aedes aegypti* (*A. aegypti*), and second less effective vector is *Aedes albopictus* (*A. albopictus*) [2–5]. Before 1970, only 9 countries suffered from dengue epidemic. However, now dengue is endemic in more than 100 countries [2, 6]. The vaccine for dengue fever has been under development, and therefore, in order to prevent the spread of dengue virus, it is important to focus on vector control [2, 7, 8].

According to Gubler, dengue epidemic in tropical and sub-tropical countries has been caused by dramatic population increase, urbanization and globalization [6]. The end of World War II era brought a rapid economic growth in many south tropic countries, which made urban area very congested. Moreover, the situation of inadequate housing and the few or no basic service such as water, sewer and waste management have created the ideal reproductive environment for mosquitoes. The crowded human communities and a large number of vectors have increased the risk of epidemic in these countries. Furthermore, globalization in recent decades enabled easy invasion of a disease. For example, airplanes coming from endemic regions have brought careers of infection to non-endemic countries.

On the other hand, cases of dengue in the non-tropical countries has been reported in past few years. In France and Croatia, the first cases occurred in 2010, and in Florida (United States) was reported in 2013 [2, 7]. Also dengue was reported in Japan after a lapse of 70 years [2]. As features of developed cities such as Tokyo, water sanitation system is well-ordered, and there are many urban green areas, which means the population of mosquitoes varies from place to place. In green area such as a park and a forest, the population of mosquitoes is large, which makes the risk of transmission of dengue high. However, in business districts and residential area, the population of mosquitoes is small, and the risk of infection is low.

In this study, we assess potential countermeasures against the dengue epidemic in a non-tropical urban city with substantial water sanitary control, using a modified Ross-Macdonald model. Here, we suppose that the non-tropical city is the region where mosquitoes are active only for a few months in a year. The Ross-Macdonald model is a mathematical model for mosquito-transmitted diseases [8], and we apply the model to the two sets of host-vector compartments that correspond to high-risk and low-risk areas. This model is a kind of two-patch metapopulation model. Metapopulation models of mosquito-transmitted diseases have been studied by several groups not only in two-patch systems [9, 10] but also in multi-patch systems [11–15], although most of those models are for malaria [10–15]. While both humans and mosquitoes are suppose to move between different patches in some models [14, 15], we assume in our model that only humans move between the two different areas. We investigate what conditions can be important for the dengue epidemic under the considered situation.

## Methods

### Model

**Fig. 1:**
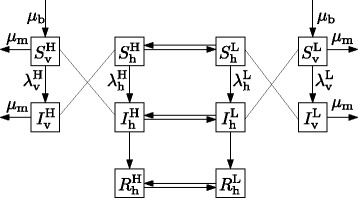
Schematic of the model. There are two sets of compartments of host and vector populations. They correspond to the high-risk (H) and low-risk (L) areas

We use a modified Ross-Macdonald model which consists of two sets of host-vector compartments (Fig. 1). Each set has susceptible-infected-recovered compartments for host and susceptible-infected compartments for vector. The two sets correspond to high-risk and low-risk areas, and only hosts can move between them. Dengue virus is classified into four serotypes, however, we here consider the situation where only one serotype is imported. The equations for the change in host and vector populations in the high-risk area are

$$\begin{array}{@{}rcl@{}} \frac{{dS}_{\mathrm{h}}^{\mathrm{H}}}{dt} &=&- \lambda_{\mathrm{h}}^{\mathrm{H}}S_{\mathrm{h}}^{\mathrm{H}} + w_{\text{HL}}S_{\mathrm{h}}^{\mathrm{L}} - w_{\text{LH}}S_{\mathrm{h}}^{\mathrm{H}},\\ \frac{{dI}_{\mathrm{h}}^{\mathrm{H}}}{dt} &=& \lambda_{\mathrm{h}}^{\mathrm{H}} S_{\mathrm{h}}^{\mathrm{H}} -\gamma I_{\mathrm{h}}^{\mathrm{H}} + w_{\text{HL}}I_{\mathrm{h}}^{\mathrm{L}} - w_{\text{LH}}I_{\mathrm{h}}^{\mathrm{H}},\\ \frac{{dR}_{\mathrm{h}}^{\mathrm{H}}}{dt} &=& \gamma I_{\mathrm{h}}^{\mathrm{H}} + w_{\text{HL}}R_{\mathrm{h}}^{\mathrm{L}} - w_{\text{LH}}R_{\mathrm{h}}^{\mathrm{H}},\\ \frac{{dS}_{\mathrm{v}}^{\mathrm{H}}}{dt} &=&- \lambda_{\mathrm{v}}^{\mathrm{H}} S_{\mathrm{v}}^{\mathrm{H}} - \mu_{\mathrm{m}} S_{\mathrm{v}}^{\mathrm{H}} +\mu_{\mathrm{b}} N_{\mathrm{v}}^{\mathrm{H}},\\ \frac{{dI}_{\mathrm{v}}^{\mathrm{H}}}{dt} &=& \lambda_{\mathrm{v}}^{\mathrm{H}} S_{\mathrm{v}}^{\mathrm{H}} - \mu_{\mathrm{m}} I_{\mathrm{v}}^{\mathrm{H}}, \end{array} $$

where *S*_h_, *I*_h_ and *R*_h_ are the populations of susceptible, infected and recovered hosts, and *S*_v_ and *I*_v_ are those of susceptible and infected vectors, respectively. The superscripts H and L represent high-risk and low-risk areas, respectively. *γ* is the host recovery rate. *w*_LH_ and *w*_HL_ are mobility rates from the high-risk area to the low-risk one and that of the opposite direction, respectively. *μ*_b_ and *μ*_m_ are the birth and death rates of vector, respectively. The forces of infection are given by

$$ \lambda_{\mathrm{h}}^{\mathrm{H}} = ab\frac{I_{\mathrm{v}}^{\mathrm{H}}}{N_{\mathrm{v}}^{\mathrm{H}}} \frac{N_{\mathrm{v}}^{\mathrm{H}}}{N_{\mathrm{h}}^{\mathrm{H}}} =ab\frac{I_{\mathrm{v}}^{\mathrm{H}}}{N_{\mathrm{h}}^{\mathrm{H}}}, \quad \lambda_{\mathrm{v}}^{\mathrm{H}} = ac(1-q)\frac{I_{\mathrm{h}}^{\mathrm{H}}}{N_{\mathrm{h}}^{\mathrm{H}}}, $$

where *a* is the biting rate, *b* and *c* are the transmission rates from vector to host and from host to vector, respectively, and *q* is the quarantine population of host. The total numbers of hosts and vectors in the high-risk area are $N_{\mathrm {h}}^{\mathrm {H}}=S_{\mathrm {h}}^{\mathrm {H}}+I_{\mathrm {h}}^{\mathrm {H}}+R_{\mathrm {h}}^{\mathrm {H}}$ and $N_{\mathrm {v}}^{\mathrm {H}}=S_{\mathrm {v}}^{\mathrm {H}}+I_{\mathrm {v}}^{\mathrm {H}}$, respectively. The equations for the change in host and vector populations in the low-risk area are

$$\begin{array}{@{}rcl@{}} \frac{{dS}_{\mathrm{h}}^{\mathrm{L}}}{dt} &=&- \lambda_{\mathrm{h}}^{\mathrm{L}}S_{\mathrm{h}}^{\mathrm{L}} + w_{\text{LH}}S_{\mathrm{h}}^{\mathrm{H}} - w_{\text{HL}}S_{\mathrm{h}}^{\mathrm{L}},\\ \frac{{dI}_{\mathrm{h}}^{\mathrm{L}}}{dt} &=& \lambda_{\mathrm{h}}^{\mathrm{L}}S_{\mathrm{h}}^{\mathrm{L}} - \gamma I_{\mathrm{h}}^{\mathrm{L}} + w_{\text{LH}}I_{\mathrm{h}}^{\mathrm{H}} - w_{\text{HL}}I_{\mathrm{h}}^{\mathrm{L}},\\ \frac{{dR}_{\mathrm{h}}^{\mathrm{L}}}{dt} &=& \gamma I_{\mathrm{h}}^{\mathrm{L}} + w_{\text{LH}}R_{\mathrm{h}}^{\mathrm{H}} - w_{\text{HL}}R_{\mathrm{h}}^{\mathrm{L}}, \\ \frac{{dS}_{\mathrm{v}}^{\mathrm{L}}}{dt} &=&- \lambda_{\mathrm{v}}^{\mathrm{L}}S_{\mathrm{v}}^{\mathrm{L}} - \mu_{\mathrm{m}} S_{\mathrm{v}}^{\mathrm{L}} + \mu_{\mathrm{b}} N_{\mathrm{v}}^{\mathrm{L}},\\ \frac{{dI}_{\mathrm{v}}^{\mathrm{L}}}{dt} &=& \lambda_{\mathrm{v}}^{\mathrm{L}}S_{\mathrm{v}}^{\mathrm{L}} -\mu_{\mathrm{m}} I_{\mathrm{v}}^{\mathrm{L}}. \end{array} $$

Here, the forces of infection are given by

$$ \lambda_{\mathrm{h}}^{\mathrm{L}} = ab\frac{I_{\mathrm{v}}^{\mathrm{L}}}{N_{\mathrm{v}}^{\mathrm{L}}} \frac{N_{\mathrm{v}}^{\mathrm{L}}}{N_{\mathrm{h}}^{\mathrm{L}}} = ab\frac{I_{\mathrm{v}}^{\mathrm{L}}}{N_{\mathrm{h}}^{\mathrm{L}}}, \quad \lambda_{\mathrm{v}}^{\mathrm{L}} = ac(1-q)\frac{I_{\mathrm{h}}^{\mathrm{L}}}{N_{\mathrm{h}}^{\mathrm{L}}}, $$

where $N_{\mathrm {h}}^{\mathrm {L}}=S_{\mathrm {h}}^{\mathrm {L}}+I_{\mathrm {h}}^{\mathrm {L}}+R_{\mathrm {h}}^{\mathrm {L}}$ and $N_{\mathrm {v}}^{\mathrm {L}}=S_{\mathrm {v}}^{\mathrm {L}}+I_{\mathrm {v}}^{\mathrm {L}}$. In order to keep the host population constant in each area, we impose a condition about the mobility rates,

(1) $$ \frac{w_{\text{HL}}}{w_{\text{LH}}} =\frac{N_{\mathrm{h}}^{\mathrm{H}}}{N_{\mathrm{h}}^{\mathrm{L}}}.   $$

### Parameters

The parameters we use is summarized in Table 1. We assume that the primary case occurs in the central part of Tokyo. The parameters about population are extracted from the data of Meguro City (Meguro-ku), which is a typical urban city in Tokyo. The population of Meguro City is about *N*_h_=2.7×10^5^ (in 2015) [16]. The high-risk area is supposed to be the area where the number density of mosquitoes is high, e.g., parks. The low-risk area is supposed to be the area where it is low, e.g., business districts and residential areas. We allocate the populations in the high-risk and low-risk areas as $N_{\mathrm {h}}^{\mathrm {H}}=N_{\mathrm {h}}\times 1/31$ and $N_{\mathrm {h}}^{\mathrm {L}}=N_{\mathrm {h}}\times 30/31$, respectively, according to the proportion of the area of parks in Meguro City [17].

**Table 1:** Definition and values of parameters in simulations

Parameter	Symbol	Value	Source
Fixed parameters			
Host population (Meguro City, Tokyo)	*N*_h_	2.7×10^5^	[16]
Host population in the high-risk (H) area	$N_{\mathrm {h}}^{\mathrm {H}}$	*N*_h_×1/31	
Host population in the low-risk (L) area	$N_{\mathrm {h}}^{\mathrm {L}}$	*N*_h_×30/31	
Transmission rate (vector to host)	*b*	0.46	[25]
Transmission rate (host to vector)	*c*	0.83	[25]
Host recovery rate	*γ*	1/7	[26]
Vector birth rate	*μ*_b_	1/30	[18]
Vector natural mortality rate	$\mu _{\mathrm {m}}^{\text {nt}}$	1/30	[18]
Uncertain parameters			
Biting rate	*a*	0.122 (base case)	
Vector excess mortality rate	$\mu _{\mathrm {m}}^{\text {ex}}$	0 (base case)	
Number of vectors per host in the H area	*r* ^*H*^	100 (base case)	[18, 19]
Number of vectors per host in the L area	*r* ^*L*^	0.22 (base case)	
Host mobility rate (from the L to H area)	*w*_HL_	0.078 (base case)	
Host mobility rate (from the H to L area)	*w*_LH_	$w_{\text {HL}}N_{\mathrm {h}}^{\mathrm {L}}/N_{\mathrm {h}}^{\mathrm {H}}$	
Quarantine proportion	*q*	0 (base case)	

The host mobility rate from the low-risk area to high-risk one *w*_HL_ for the base case is estimated from the number of visitors to parks [16]. That of the opposite direction *w*_LH_ is given by Eq. (1).

The number of vectors per host in the high-risk area, $r^{H}=N_{\mathrm {v}}^{\mathrm {H}}/N_{\mathrm {h}}^{\mathrm {H}}$, for the base case is evaluated from the biting density in a park in urban Tokyo and the attraction rage of a human bite [18, 19]. That in the low-risk area, $r^{L}=N_{\mathrm {v}}^{\mathrm {L}}/N_{\mathrm {h}}^{\mathrm {L}}$, is given so that the basic reproduction number for an isolated low-risk area

$$ R_{0}^{\mathrm{L}} = \sqrt{\frac{a^{2}bc(1-q)r^{\mathrm{L}}}{\mu_{\mathrm{m}}\gamma}} $$

is about 0.5.

**Fig. 2:**
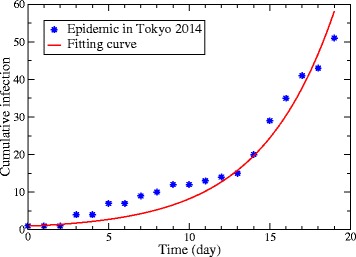
Cumulative number of infected hosts at early times. The fitting curve is the result for estimation of the biting rate in the base case. It is fitted to the cumulative cases for the initial 19 days of the dengue epidemic in Tokyo 2014 [20]

The biting rate *a* for the base case is estimated by fitting to the data of the dengue epidemic in Tokyo 2014 [20]. Since the closure of a park was started after 19 days from the index case of the epidemic [21], the curve of the cumulative number of infected hosts is fitted to the cumulative cases during 19 days by a least square method. The result of the fitting is illustrated in Fig. 2.

### Countermeasures

We assess five potential countermeasures (Table 2): (1) restriction of the travel between the high-risk and low-risk areas, (2) insecticide application after the outbreak, (3) call on citizens to use mosquito repellents, (4) reducing carrying capacity of mosquitoes and (5) isolation of infected people. Measures 1 and 2 are implemented after 7 days from the primary case. For Measure 1, the mobility rates *w*_HL_ and *w*_LH_ change to take another value from the 7th day to the 90th day. The vector mortality rate is the sum of the natural mortality rate $\mu _{\mathrm {m}}^{\text {nt}}$ and the excess mortality rate $\mu _{\mathrm {m}}^{\text {ex}}$. For Measure 2, the excess mortality rate $\mu _{\mathrm {m}}^{\text {ex}}$ takes a nonzero value during a certain period from the 7th day. Measure 3 is relevant to the biting rate *a*. When people use mosquito repellents, the biting rate is expected to decrease. Measure 4 is relevant to the numbers of vectors per host, *r*^H^ and *r*^L^. Measure 5 is relevant to the quarantine proportion *q*. Here, isolation means to keep an infected person from mosquitoes.

**Table 2:** Potential countermeasures

	Countermeasure	Implementation period
(1)	Restriction of the travel between the high-risk and low-risk areas	from the 7th day to the end
(2)	Insecticide application after the outbreak	a certain period from the 7th day
(3)	Call on citizens to use mosquito repellents	entire period
(4)	Reducing carrying capacity of mosquitoes	entire period
(5)	Isolation of infected people	entire period

Here, we note that each of populations in the high-risk and low-risk areas does not change with time. In other words, some people live in the high-risk area, and the others live in the low-risk area. Measure 1 is to reduce the traffic between the high-risk and low-risk areas. Although the parameters for the high-risk area are taken as those for parks, the high-risk area is not limited to parks. The high-risk area includes the area where the sanitary control of water is not substantial.

### Seasonality

**Fig. 3:**
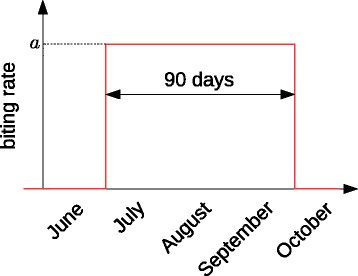
Seasonality of vector activity. The period when mosquitoes are active is taken to be 90 days in summer. The parameters such as the biting rate is assumed to be constant during the period

In Tokyo today, mosquitoes are active only in summer and cannot be alive in winter. In this paper, we assume that mosquitoes are active for only 90 days in summer and that their activity is constant during the period as illustrated in Fig. 3.

## Results and discussion

### Basic reproduction number

The basic reproduction number is given by

(2) $$\begin{array}{@{}rcl@{}} R_{0} = \sqrt{\frac{a^{2} bc(1-q)}{\mu_{\mathrm{m}} \gamma}} \sqrt{\frac{X+\sqrt{Y^{2} + 4Z}}{2(\gamma + w_{\text{HL}} + w_{\text{LH}})}},  \end{array} $$

where

$$\begin{array}{@{}rcl@{}} X &=& r^{\mathrm{H}}(\gamma + w_{\text{HL}}) + r^{\mathrm{L}}(\gamma + w_{\text{LH}}),\\ Y &=& r^{\mathrm{H}}(\gamma + w_{\text{HL}}) - r^{\mathrm{L}}(\gamma + w_{\text{LH}}),\\ Z &=& r^{\mathrm{H}}r^{\mathrm{L}}w_{\text{HL}}w_{\text{LH}}. \end{array} $$

**Fig. 4:**
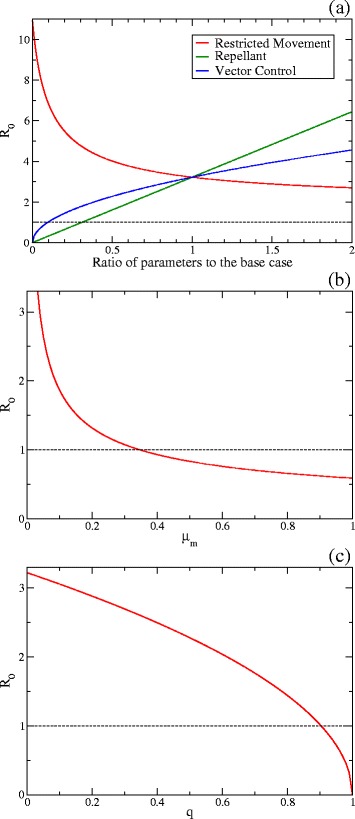
Sensitivity analysis of the basic reproduction number *R*_0_. **a** *R*_0_ as the function of the ratio of each parameter to each value of the base case. The parameters are the host mobility rates *w*_HL_ and *w*_LH_, which are relevant to Measure 1 (restricted movement), for the *red curve*, the biting rate *a*, which is relevant to Measure 3 (use of repellents), for the *green one*, and the numbers of vectors per host *r* ^H^ and *r* ^L^, which are relevant to Measure 4 (vector control), for the *blue one*. **b** *R*_0_ as the function of the vector mortality rate *μ*_m_, which is relevant to Measure 2 (insecticide application). **c** *R*_0_ as the function of the quarantine proportion *q*, which is relevant to Measure 5 (isolation)

The sensitivity analysis of *R*_0_ is shown in Fig. 4. In Fig. 4(a), *R*_0_ is plotted as the function of the ratio of each parameter to each value of the base case for Measures 1 (restricted movement), 3 (use of repellents) and 4 (vector control). Reduction of the biting rate and the number of mosquitoes per host reduce *R*_0_ as illustrated by the green and blue curves. Since *R*_0_ is linearly proportional to the biting rate, Measure 3 is especially effective.

The red curve in Fig. 4(a) implies that *R*_0_ increases as the mobility rate becomes small. Actually, Eq. (2) is nearly equal to the basic reproduction number for an isolated high-risk area when *w*_HL_=*w*_LH_=0 since *r*^H^≫*r*^L^ and *r*^L^ is negligible. In other words, the restricted movement effectively increases the number density of vectors in the high-risk area.

In Figs. 4(b) and (c), *R*_0_ is plotted as the functions of the vector mortality rate *μ*_m_ and the quarantine proportion *q*, which are relevant to Measures 2 and 5, respectively. *R*_0_ decreases when *μ*_m_ or *q* increases.

### Cumulative number of infected hosts

**Fig. 5:**
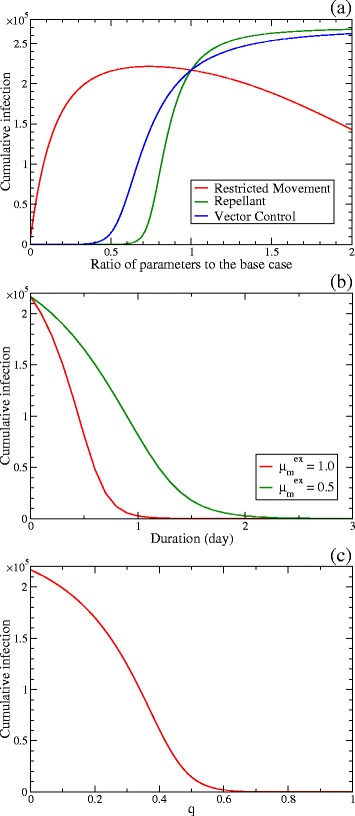
Sensitivity analysis of the cumulative number of infected hosts. The cumulative numbers of infected hosts after 90 days are plotted. **a** Plotted as the function of the ratio of each parameter to each value of the base case. The parameters are the host mobility rates *w*_HL_ and *w*_LH_ after 7 days, which are relevant to Measure 1 (restricted movement), for the *red curve*, the biting rate *a*, which is relevant to Measure 3 (use of repellents), for the *green one*, and the numbers of vectors per host *r* ^H^ and *r* ^L^, which are relevant to Measure 4 (vector control), for the *blue one*. **b** Plotted as the function of the implementation period of Measure 2 (insecticide application). During the insecticide application, the vector excess mortality rates are $\mu _{\mathrm {m}}^{\text {ex}}=1.0$ and 0.5 for the red and green curves, respectively. **c** Plotted as the function of the quarantine proportion *q*, which is relevant to Measure 5 (isolation)

The sensitivity analysis of the cumulative number of infected hosts for 90 days is shown in Fig. 5. In Fig. 5(a), the cumulative number of the infected is plotted as the function of the ratio of each parameter to each value of the base case for Measures 1 (restricted movement), 3 (use of repellents) and 4 (vector control). As illustrated by the green and blue curves, the reduction of the biting rate and the number of vectors per host substantially reduce the cumulative number of the infected for 90 days even when *R*_0_>1.

Although *R*_0_ decreases monotonically with the mobility rates as shown in Fig. 4(a), the cumulative number of the infected for 90 days, i.e. the red curve in Fig. 5(a), has a maximum. When the mobility rates are large, the growth of infection is slow because the effective number density of vectors becomes small. Thus, a relatively small number of people are infected for 90 days. When the mobility rates are small enough, the growth of infection becomes fast in the high-risk area, however, the epidemic in the low-risk area does not immediately follow that in the high-risk area, and the cumulative number of the infected diminishes. Thus, the maximum of the cumulative number of the infected for 90 days appears at a moderate mobility rate. We discuss the details of effects of Measure 1 (restricted movement) later in the next subsection.

In Fig. 5(b), the cumulative number of the infected is plotted as the function of the implementation period of Measure 2 (insecticide application). When the excess mortality rate $\mu _{\mathrm {m}}^{\text {ex}}=1.0$, only one day is effective enough to decrease the number of the infected substantially. When the excess mortality rate is half, the period to obtain the same result doubles.

In Fig. 5(c), the cumulative number of the infected is plotted as the function of the quarantine proportion *q*, which is relevant to Measure 5 (isolation). Although *R*_0_<1 for *q*>0.9 as shown in Fig. 4(c), the cumulative number of the infected for 90 days is small enough for *q*>0.6.

### Effects of restricted movement

**Fig. 6:**
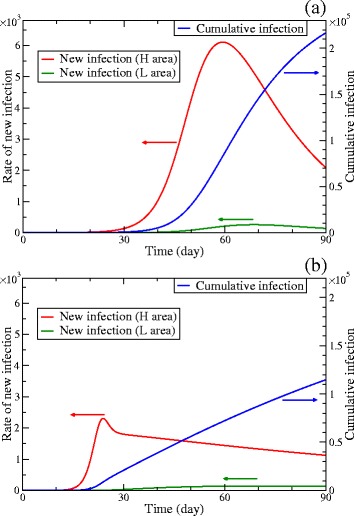
Time dependence of the rates of new infections of hosts and the cumulative number of infected hosts. The cumulative infection (*the right axis*) and the rates of new infections (*the left axis*) in the high-risk (H) and low-risk (L) areas are plotted as the functions of time. **a** The base case. **b** The case where Measure 1 is implemented: The values of the mobility rates *w*_HL_ and *w*_LH_ are down to 1/10 of the base case after 7 days

The cumulative number of infected hosts for 90 days decreases when the mobility rates are sufficiently small, however, the epidemic starts earlier than the base case. That is illustrated in Fig. 6. The time dependence of the cumulative number of the infected in the base case is shown as the blue curve in Fig. 6(a). It starts to increase noticeably after 30 days. However, the blue curve in Fig. 6(b), where the mobility rates are 1/10 of the base case, starts to increase noticeably after 20 days.

The red curves in Fig. 6 show that the early start of the epidemic is induced by the epidemic in the high-risk area. The red and green curves are the rates of new infections of hosts in the high-risk and low-risk areas, respectively. In the base case, Fig. 6(a), the difference in day on which the peak of new infections appears is rather small between the two areas. However, in Fig. 6(b), where the mobility rates are 1/10 of the base case, the peak of the new infections in the high-risk area is more than 40 days earlier than that in the low-risk area.

Effects of restricted movement are summarized as follows. First, the effectively high number density of vectors accelerates the epidemic in the high-risk area. Then, a certain amount of infected hosts moves to the low-risk area although the mobility rates are small. Consequently, the epidemic starts early, while the growth of infections is slow.

### Effects of a core population

The nontrivial results for Measure 1 (restricted movement) is caused by the heterogeneous populations which are divided into the high-risk and low-risk areas. When the mobility rates between the two areas are small, the population in the high-risk area is a kind of core population. It is often suggested that targeting a core population is the key to prevent the spread of disease. For example, several works on sexually transmitted diseases suggested control or treatment targeting core members, who are highly active in sexual behavior, should be better than targeting the whole population [22, 23].

The earlier peak of new infections in the high-risk area than in the low-risk area, which is shown in Fig. 6(b), is also one of the effects caused by the core population. The cascade of infection from core to other populations is observed also in other diseases. For example, in the case of pandemic influenza, the burden of disease shifts from children to adults [24]. This means that new infections in the core population, which has many contacts, occur earlier than in the other population.

Heterogeneity is essential to form a core population. Although the effect of heterogeneity is often discussed in studies of metapopulation models, formation of a core population has not attracted attention in metapopulation models for mosquito-transmitted diseases. Lee and Castillo-Chavez assessed the dengue transmission dynamics in heterogeneous environment using a two-patch model [9]. In their study, however, the local basic reproduction number of each patch is taken to be larger than 1 in the base case, which is different from our situation. In studies of two-patch malaria models [10, 15], it is indicated that human movement can cause the persistence of malaria in a patch with a local basic reproduction number below 1. Although the situation is similar to ours, their focus is on the endemic condition caused by human movement. In contrast, our results for Measure 1 (restricted movement) give an insight that the restriction of human movement can sometimes cause a larger-size epidemic than that of a no-restriction case.

### Limitations

We consider only one serotype of dengue virus in the above. It is unlikely that several serotypes are imported at the same time into a non-tropical region. However, if epidemics of several serotypes were took into account, the model should be changed into a more complicated model.

In this paper, particular seasonality is assumed: Mosquitoes keep constant activity only for 90 days in a year. Since the parameter values are taken for the worst case, the results may be overestimated. This limitation is the compensation for the simplicity of the model. The length of the period when mosquitoes are active affects the cumulative number of infected hosts but not the basic reproduction number.

## Conclusions

We have assessed the five potential countermeasures against the dengue epidemic in a non-tropical urban city such as Tokyo, using a mathematical model in which compartments are divided into the high-risk and low-risk areas. Measures 2 (insecticide application), 3 (use of repellents), 4 (vector control) and 5 (isolation) are more or less effective, and substantial reduction of the cumulative number of infected hosts for 90 days can be expected for a small *R*_0_ even if *R*_0_>1. However, Measure 1 (restricted movement) can have the opposite effect, depending on the mobility rates. Reduction of the mobility rates, namely, the restriction of travel between the high-risk and low-risk areas, results in the formation of a kind of core population. The epidemic in the core population (in the high-risk area) can promote that in the entire population when there are moderate contacts between them.
